# Spontaneous Recovery of Fear Reverses Extinction-Induced Excitability of Infralimbic Neurons

**DOI:** 10.1371/journal.pone.0103596

**Published:** 2014-08-04

**Authors:** Emmanuel Cruz, Ana V. López, James T. Porter

**Affiliations:** Department of Physiology and Pharmacology, Ponce School of Medicine, Ponce, Puerto Rico; Radboud University, Netherlands

## Abstract

In rodents, the infralimbic (IL) region of the medial prefrontal cortex plays a key role in the recall of fear extinction. Previously we showed that fear conditioning decreases the intrinsic excitability of IL neurons, and that fear extinction reverses the depressed excitability. In the current study, we examined the time course of the extinction-induced changes in adolescent rats. Immediately after extinction, IL neurons continued to show depressed excitability. However 4 hours after extinction, IL neurons showed an increase in evoked spikes that correlated with a reduced fast afterhyperpolarizing potential. This suggests that acquisition of fear extinction induces an increase in spike firing 4 hours later, during the consolidation of extinction. We also examined IL excitability in a group of rats that showed spontaneous recovery of fear 17 days after extinction (SR group). Similar to neurons after fear conditioning, IL neurons from the SR group showed depressed intrinsic excitability compared to neurons 4 hours after extinction, suggesting that extinction-induced enhancement in intrinsic excitability decreases with time reverting back to a depressed state. These results suggest that plasticity in IL contributes to the spontaneous recovery of fear and preventing this depression of IL excitability could prolong fear extinction.

## Introduction

Learning and remembering which environmental cues predict danger is critical for survival. However, it is equally important to recognize when cues no longer signal danger to avoid unnecessarily restricting behaviors such as foraging for food. This shift in behavioral response to cues is modeled in the laboratory as extinction of conditioned fear. It is well accepted that the infralimbic region (IL) of the medial prefrontal cortex (mPFC) plays a critical role in reducing conditioned fear responses during the recall of extinction [33]. During the recall of extinction, IL neurons fire in response to a conditioned tone and show increased activity, [3], [9], [17]. Although these studies indicate that fear extinction induces changes in IL, what cellular changes occur and the time course of these cellular changes are still being elucidated.

Previously we showed that fear conditioning decreases the intrinsic excitability of IL neurons and that extinction reverses the depressed IL excitability [29]. These results suggest that one of the mechanisms contributing to the increased activation of IL during extinction recall is an increase in the intrinsic excitability of IL neurons. However, this initial study examined the cellular excitability of IL neurons at a single time point, 24 hours after acquisition of extinction. We interpreted the increase in excitability to mean that consolidation of extinction modified the intrinsic excitability of IL neurons. However, we did not determine whether these intrinsic changes occurred during the acquisition of extinction or later during the consolidation of the extinction memory. Furthermore, it is also possible that the recall of extinction actually induced the intrinsic changes.

With the passage of time after fear extinction, the fear response spontaneously recovers [2], [23], [26]. Given the importance of IL activity for the recall of fear extinction [17], [18], [32], the spontaneous return of the conditioned fear response could be secondary to the loss of extinction-induced plasticity in IL. However, it is equally possible that plasticity in IL is not involved in spontaneous recovery and that instead the plasticity responsible for the spontaneous recovery of fear occurs in other structures in the extinction circuit such as the amygdala, hippocampus, or periaqueductal grey [7], [19], [20].

In this study, we examined the time course of the extinction-induced changes in IL intrinsic excitability to address the following questions: Do extinction-induced changes in IL excitability occur during the acquisition phase or consolidation phase? Do these changes reverse with the spontaneous recovery of fear?

## Materials and Methods

### Behavioral procedures

The procedures were approved by the Institutional Animal Care and Use Committee (IACUC) of the Ponce School of Medicine in compliance with NIH guidelines for the care and use of laboratory animals and the 2013 American Veterinary Medical Association Guidelines for the Euthanasia of Animals. Male Sprague-Dawley rats from the Ponce School of Medicine colony were maintained on a 12/12 hr light/dark schedule with free access to food and water.

On day 1, all rats (∼P30 during fear conditioning) received 3 pairings of a 30 s tone (4 kHz, 80 dB) that co-terminated with a 0.4 mA scrambled footshock, 0.5 sec in duration (Conditioning phase). After matching for equivalent levels of freezing, rats were divided into a Conditioned group (COND), an Extinction immediate sacrifice group (EXT-0), an Extinction 4 hour sacrifice group (Ext-4), and a Spontaneous Recovery group (SR). On day 2, rats in the COND group were given one test tone and sacrificed at the same time as the Ext-0 and Ext-4 groups. On day 2, rats in the EXT-0, Ext-4, and SR groups were given 12 tone-alone trials in the conditioning chamber (Extinction phase). Rats in the EXT-0 and Ext-4 groups were respectively sacrificed immediately after extinction and 4 hours after extinction. Rats spent the 4 hr delay in their home cages. On day 3, the SR group received two test tones in the same chamber to test for recall of extinction (Test phase). After 16 days in their home cages, SR rats were given 2 tone-alone trials to access their recall of extinction and then immediately sacrificed. During all phases the average interval between tones was 2 min.

### Current-clamp recordings

Rats were anesthetized with pentobarbital (150 mg/kg) and perfused through the heart with ice cold sucrose solution: 252 mM sucrose, 2 mM KCl, 1.25 mM NaH_2_PO_4_, 3 mM MgSO_4_, 26 mM NaHCO_3_, 20 mM glucose and 1 mM CaCl_2_. Brains were placed in ice cold artificial cerebral spinal fluid (ACSF) containing 126 mM NaCl, 3 mM KCl, 1.25 mM NaH_2_PO_4_, 1 mM MgSO_4_, 26 mM NaHCO_3_, 20 mM glucose and 2 mM CaCl_2_ and bubbled with 95% O_2_ and 5% CO_2_. Prefrontal slices (300 µm) were cut and incubated in room temperature ACSF containing the NMDA receptor blocker MK-801 (10 µM).

Recordings were performed blind with respect to group. Whole-cell current-clamp recordings were obtained from IL pyramidal neurons located in layers II/III and V with glass pipettes (3–5 MΩ) filled with a solution containing 150 mM KMeSO_4_, 10 mM KCl, 0.1 mM EGTA, 10 mM HEPES, 0.3 mM GTP, and 0.2 mM ATP (pH 7.3, 291 mOsm). To examine the effects of training on membrane excitability, neurons were manually held at −70 mV and injected with 800 ms depolarizing current pulses ranging from −40 to 350 pA in 10 pA increments with an intertrial interval of 500 ms. Responses were filtered at 4 kHz, digitized at 10 kHz, and saved using pCLAMP9 (MultiClamp 700A, Axon Instruments, Union City, CA). Membrane potentials were not corrected for the junction potential of 9 mV. The number of action potentials evoked by each current intensity was counted from individual responses. The series resistance (Ra) was equal across groups (Table 1). The input resistance (R_in_) was measured from a 5 mV, 50 ms depolarizing pulse in voltage-clamp mode. The fast afterhyperpolarizing potential (fAHP) was measured as the peak between the second and third spike from the trace giving the maximum number of spikes by subtracting the voltage at the peak of the fAHP from the threshold potential for spike initiation. The slow afterhyperpolarizing potential (sAHP) was measured as the average potential during a 50 ms period beginning 280 ms after the end of the depolarizing pulse [29] in traces with the same number of spikes (2 spikes).

**Table 1:** Electrophysiological Properties of IL Neurons.

	COND (n = 34)	EXT-0 (n = 22)	EXT-4 (n = 18)	SR (n = 18)	Naïve P50 (n = 15 )
Vm (mV)	−60±1	−62±1	−62±1	−62±2	−60±1
R_in_ (MΩ)	254±16	218±17	256±20	327±30	254±15
Ra (MΩ)	17±1	18±1	18±1	20±1	18±1
Rheobase (pA)	103±10	111±11	102±12	74±10	94±12
Threshold (mV)	−37±1	−32±1*	−38±1	−36±1	−37±1
mAHP (mV)	−4.4±0.3	−4.5±0.4	−3.4±0.3	−4.0±0.4	−4.8±0.6
sAHP (mV)	−2.7±0.3	−3.0±0.4	−2.3±0.4	−3.3±0.3	−2.8±0.4

*One-way ANOVA showed a main effect of group (F(3,88)  = 5.84, p = 0.001). Post-hoc comparisons indicated EXT-0 group had a more positive threshold than the COND (p = 0.004) and EXT-4 (p = 0.001) groups.

Neurons were labeled with 5 mM biocytin and visualized with a standard avidin-biotin peroxidase procedure (Vectastain ABC kit, Vector Laboratories, Burlingame, CA). Neurons that were not located in IL, or that were not pyramidal-shaped with obvious apical dendrites, were excluded from the analysis.

### Statistical analysis

Conditioned fear was measured as the percent of time spent freezing during the 30 sec tone with no movement except respiration (FreezeScan, Clever Systems). The electrophysiological data were analyzed using Clampfit (Axon Instruments, Union City, CA). Behavioral and electrophysiological data were compared with Student's t-test, paired t-test, or one-way ANOVA (STATISTICA, Statsoft, Tulsa, OK). Following a significant main effect with a one-way ANOVA, post-hoc tests were performed with Tukey HSD test or Fisher LSD (spikes evoked by increasing depolarizing pulses). Values are reported as the mean ± the standard error of the mean (S.E.M.).

## Results

### Spontaneous recovery reverses the extinction-induced increase in IL excitability

To examine the time course of the intrinsic excitability changes of IL neurons after fear extinction, we made whole-cell patch-clamp recordings of IL neurons from four groups of rats. A conditioned group (COND, n = 12) received fear conditioning (3 tone-shock pairings) on day 1, a recall tone on day 2, and were sacrificed. Two extinction groups received fear conditioning on day 1 and extinction (12 tones without shocks) on day 2, and were sacrificed either immediately (EXT-0, n = 7) or 4 hours later (EXT-4, n = 6). A spontaneous recovery (SR n = 7) group received fear conditioning on day 1, extinction on day 2, and recall of extinction (2 tones without shocks) on day 3. After 16 days in their home cages, the SR group was tested for spontaneous recovery on day 20 (2 tones without shocks) and sacrificed.

**Figure 1:**
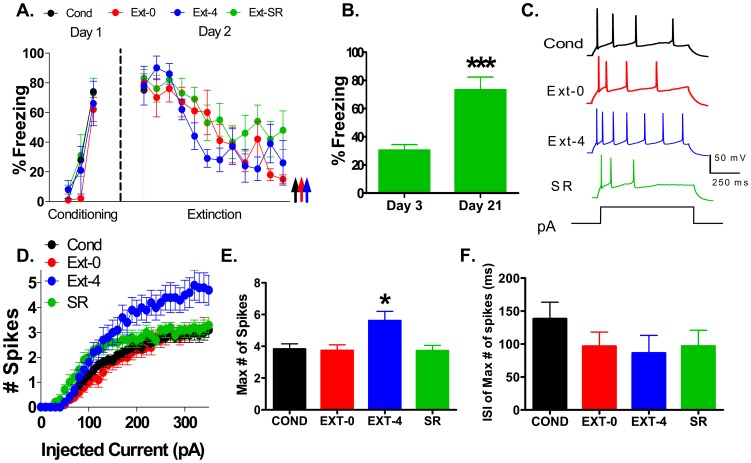
The intrinsic excitability of IL pyramidal neurons increased within 4 hours after extinction and reversed with the spontaneous recovery of fear. A. Percent of time that rats spent freezing to the tone in groups that were conditioned (Cond; n = 13 rats, black), and fear extinguished (Ext-0; n = 9 rats, red; Ext-4; n = 6 rats, blue; SR; n = 8 rats, green). The Ext-0 group was sacrificed immediately after extinction, and the Ext-4 group was sacrificed 4 hours after extinction. B. Average freezing of the SR group on day 3 and day 21 to two tones. SR rats were sacrificed immediately after testing on day 21. C. Sample traces showing responses of IL neurons from each group to a 200 pA depolarizing current step. D. Number of spikes evoked in IL neurons by increasing depolarizing steps in the Cond (n = 34), Ext-0 (n = 22), Ext-4 (n = 18), and SR (n = 18) groups. E. Average maximum number of spikes evoked in each group. F. Average first interspike interval (ISI). ***p<0.001, *p<0.05.

Figure 1A shows the average tone-induced freezing for all groups. On day 1, rats in the COND, EXT-0, EXT-4, and SR groups acquired similar levels of conditioned freezing (COND: 74%; EXT-0: 62%; EXT-4: 66%; SR: 73%: F(3,28)  = 0.36, p = 0.78). On day 2, all groups showed similar levels of freezing to the first tone (COND: 75%; EXT-0: 80%; EXT-4: 78%; SR: 83%: F(3,28)  = 0.22, p = 0.88) indicating good recall of the conditioned fear. The EXT-0, EXT-4, and SR groups showed good extinction with a gradual decrease in fear across the 12 trials (EXT-0: t(6)  = 2.45, p<0.001; EXT-4: t(5)  = 2.57, p = 0.03, and SR: t(6)  = 2.45, p = 0.05 compared to the first tone). As shown in Figure 1B, rats of the SR group showed good extinction recall on day 3 (30%), and spontaneous recovery of fear on day 21 (73% t(6)  = 2.45, p = 0.001 compared to extinction recall).

After sacrifice, the excitability of IL neurons was examined with whole-cell patch-clamp recordings in brain slices. Neurons were labeled with biocytin and later confirmed to be IL pyramidal neurons located in layers II/III or V. To assess the intrinsic excitability, we recorded the number of action potentials evoked by depolarizing current steps (Figure 1C–D). One-way ANOVA revealed a main effect of group at all intensities between 230 and 350 pA (all comparisons p≤0.03). Post-hoc comparisons indicated that IL neurons from the COND group fired the same number of spikes in response to the depolarizing pulses as neurons from the EXT-0 (p≥0.50) and SR (p≥0.45) groups. In contrast, neurons from the EXT-4 group fired more spikes than the COND (p≤0.02) and EXT-0 (p≤0.02) groups. At all intensities from 240 to 350 pA except 270 pA (p = 0.07), the EXT-4 group fired more spikes than the SR group (p≤0.04). We also measured the maximum number of spikes that could be elicited by any size current pulse for each cell (Figure 1E). The average maximum number of spikes was significantly increased in the Ext-4 group by approximately 50% compared to the COND, EXT-0, and SR groups (COND: 3.8 spikes; EXT-0: 3.6 spikes; EXT-4: 5.6 spikes; SR: 3.7 spikes). One-way ANOVA showed a main effect of group (F(3,88)  = 4.50, p = 0.006) and post-hoc comparisons indicated that the average maximum number of spikes of the EXT-4 group was higher than the COND (p = 0.013), EXT-0 (p = 0.010), and SR (p = 0.02) groups. The EXT-0 and the SR groups were not different from COND group (p = 0.99, p = 1.00).

Since we also found a decrease in the first interspike interval (ISI) of IL neurons 24 hours after extinction [29], we examined the four groups for differences in the first ISI which was measured as the interval between the first 2 spikes from the trace giving the maximum number of spikes (Figure 1F). One-way ANOVA showed no main effect of group (F(3,88)  = 0.99, p = 0.40) indicating that all four groups had similar average first ISIs (COND: 138 ms; EXT-0: 104 ms; EXT-4: 86 ms; SR: 97 ms). This finding suggests that the intrinsic IL bursting does not increase until 24 hours after extinction [29].

Despite the difference in spike count, there was no difference in the average intensity needed to evoke a single spike (rheobase), resting membrane potential (V_m_), or the input resistance (R_in_; Table 1). However, we did find an increase in the spike threshold in the Ext-0 group (Table 1). Taken together these findings indicate that the intrinsic excitability of IL neurons increases during the consolidation phase of extinction and reverses with the spontaneous recovery of fear.

### Spontaneous recovery reverses the decrease in fAHP induced by fear extinction

**Figure 2:**
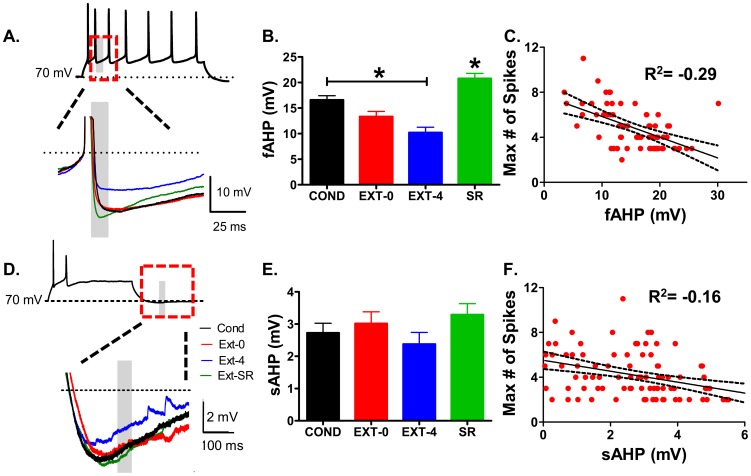
Spontaneous recovery reverses the decrease in fAHP induced by fear extinction. A. Sample traces for each group showing fAHP measured within gray bar. Traces have been aligned to overlap at the spike threshold. B. Average of the fAHP for each group. C. Correlation between maximum number of spikes and fAHP for all neurons. D. Sample traces showing sAHP measured within gray bar. E. Average of the sAHP for each group. F. Lack of correlation between number of spikes and sAHP for all neurons. *p<0.05.

Since the fAHP and sAHP in IL neurons is reduced 24 hours after extinction [29], we examined the fAHP and sAHP in all groups. The fAHP was reduced 4 hours after extinction and reversed with spontaneous recovery of fear (Figure 2A–B). One-way ANOVA showed a main effect of group (F(3,65)  = 19.91, p<0.001). Post-hoc comparisons indicated that the COND group had a larger fAHP than the EXT-4 (p<0.001) group with a trend towards being larger than the EXT-0 (p = 0.07) group (COND: 16 mV; EXT-0: 13 mV; EXT-4: 10 mV; SR: 21 mV). SR reversed the decreased fAHP observed 4 hours after extinction (P<0.001). Further analysis of all the neurons in the COND, EXT-0, EXT-4, and SR groups (Figure 2C) showed a strong inverse correlation between the maximum number of spikes and fAHP (R^2^ = −0.29).

In contrast to the fAHP, there was no difference in the sAHP among the groups (F(3,88)  = 1.08, p = 0.36, Figure 2D–E) suggesting that the reduced sAHP seen 24 hours after extinction [29] occurred between 4 and 24 hours after extinction and reversed with spontaneous recovery of fear. Consistent with the lack of change in the sAHP, the maximum number of spikes was not correlated with sAHP (R^2^ = −0.16; Figure 2F). As found previously [29], there was also no difference in the medium afterhyperpolarization (mAHP; Table 1).

### IL excitability of SR group is depressed compared to age-matched controls

**Figure 3:**
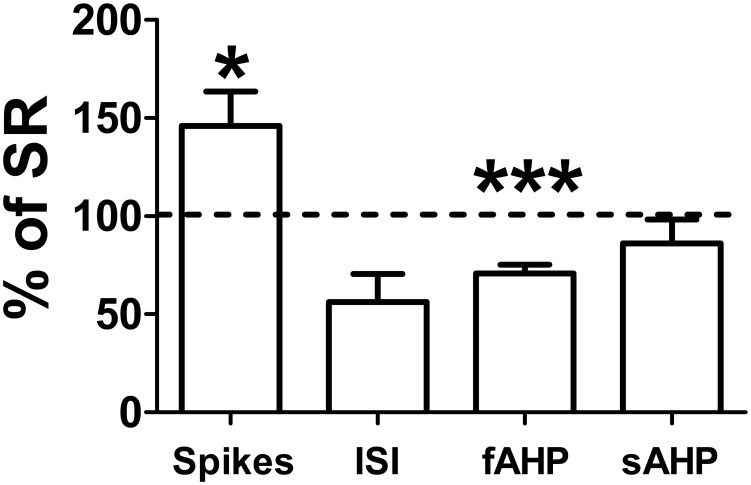
IL neurons of naïve age-matched rats are more excitable than IL neurons of the SR rats. The number of spikes, ISI, fAHP, and sAHP of IL neurons (n = 15) of P50 naïve rats (n = 5) are shown normalized to the average values of the SR group. Dashed line indicates values of SR group.

Since the SR rats were 50 days old at the time of sacrifice, they were about 20 days older than the rats in the other groups. To test whether the age of the animals in the SR group affected their IL intrinsic excitability, we compared the excitability of the SR group to an age-matched untrained naïve group (NP50, n = 5 rats). As shown in Figure 3, IL neurons (n = 15) of the NP50 group fired significantly more spikes than the SR group (NP50: 5.4 spikes; SR: 3.7 spikes; t(31)  = 2.04, p = 0.02). In addition, the fAHP was smaller in the NP50 group compared to the SR group (NP50: 15 mV; SR: 21 mV; t(26)  = 2.06, p<0.001). No difference was observed in the first ISI (NP50: 54 ms; SR: 97 ms; t(31)  = 2.04, p = 0.16) or in the sAHP (NP50: 2.8 mV; SR: 3.3 mV; t(31)  = 2.04, p = 0.38). These results indicate that the SR group was depressed compared to age-match animals. Therefore, the depressed excitability found in the SR group was not due to the age of the animals.

## Discussion

In this study, we examined how quickly IL intrinsic excitability increases after acquisition of auditory fear extinction and whether IL excitability decreases with the spontaneous recovery of fear. Our main findings are: (1) four hours after fear extinction the number of spikes fired increased and the fAHP decreased and (2) the increase in IL intrinsic excitability was lost with the spontaneous recovery of fear. These findings suggest that the intrinsic excitability of IL neurons increases during consolidation of extinction and reverses to a depressed state with the spontaneous recovery of fear.

Since our experiments used adolescent rats, it is possible that similar intrinsic changes do not occur in adults. However, although adolescent rats show slower extinction learning [12], [14], they do show fear extinction [1], [11], [12], [14], [29] and there is no evidence that fear extinction in adolescent and adult animals involves different mechanisms. In fact, fear extinction at both ages requires activation of IL [28], [32], mGluR5 signaling [6], [31], ERK in IL [12], intercalated cells in the amygdala [1], [13], and NMDA receptors [3], [14]. Therefore, our findings in adolescent rats are likely to also occur in adult rats as well.

The plasticity in IL that contributes to extinction recall appears to initiate during the acquisition of extinction and continues for several hours after the final exposure to the conditioned stimulus [3]. Immediately after extinction training, single-unit recordings in vivo show an increase in IL burst firing [3] which may be mediated by synaptic or neuromodulatory effects rather than intrinsic changes. The intrinsic excitability of IL neurons did not increase until 4 hours after extinction suggesting that a time-dependent molecular process such as protein synthesis contributes to the increased intrinsic excitability of IL neurons. Consistent with this possibility, consolidation of the extinction memory requires protein synthesis in IL [27]. Furthermore, both consolidation of extinction and the intrinsic changes in IL require activation of mGluR5 [6], [31], a known driver of protein synthesis [5]. Taken together, these findings suggest that mGluR5 activation during extinction initiates a cascade leading to a protein synthesis-dependent increase in IL intrinsic excitability.

Our results further suggest that different ion channels may mediate the increased number of evoked spikes and increased bursting. The initial increase in number of spikes 4 hours after extinction temporally correlates with a reduction in the potassium currents underlying the fAHP, whereas intrinsic burst firing did not increase until 24 hours later along with a reduced sAHP [29]. Although the temporal separation of the intrinsic changes suggests that they are mediated by two different mechanisms, both intrinsic changes depend on signaling via mGluR5 receptors [6], [31]. It is also important to note that since some rats fail to recall fear extinction despite showing good extinction learning [3], our EXT-0 and EXT-4 findings may be contaminated by rats that failed to consolidate extinction. Therefore, some excitability changes may have occurred earlier after extinction than detected.

Many studies have examined the mechanisms necessary to consolidate fear extinction memory and prevent the rapid spontaneous recovery of fear 24 hours later (reviewed in [25], however much less is known about the structures and cellular mechanisms that mediate the spontaneous recovery of conditioned fear after consolidation of extinction. The use of cfos expression suggests that decreased neuronal activity in IL contributes to the spontaneous recovery of conditioned taste aversion [15]. In a similar manner, our findings suggest that depressed IL intrinsic excitability leads to the spontaneous recovery of conditioned fear by making IL neurons less responsive to hippocampal [10], thalamic [8], or amygdala inputs [34]. In support of this possibility, field potentials in the mPFC evoked by ventral hippocampal stimulation were depressed with the spontaneous recovery of fear [10]. Interestingly, fear extinction also reduces the fAHP in IL-projecting neurons in the amygdala [30], raising the possibility that a parallel increase in the fAHP in these neurons could contribute to spontaneous recovery. It is also possible that weakening of glutamatergic synapses strengthened by fear extinction [1], [4], [22], [31] may contribute to the spontaneous recovery of fear.

**Figure 4:**
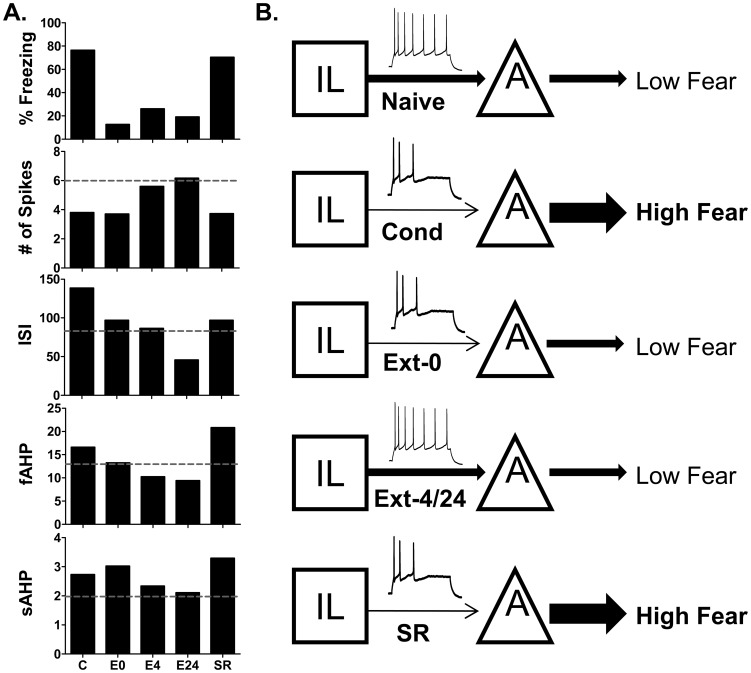
Summary and model of the changes in IL intrinsic excitability at different behavioral time-points. A. Average values of the measured intrinsic parameters. C =  COND, E0 =  EXT-0, E4 =  EXT-4, E24  = 24 hours after extinction, SR  =  Spontaneous Recovery. Dash-line indicates values of naïve group. Naïve and E24 values used with permission from the Journal of Neuroscience (Santini et al., 2008). B. Schematic representation of IL and amygdala interactions at different behavioral time-points.

Figure 4A incorporates our previous findings [29] with those of the current paper to summarize the time course of the intrinsic changes in IL from fear conditioning, extinction, and spontaneous recovery of fear. From the summary, it is clear that the number of evoked spikes in IL neurons closely follows fear expression such that when fear is high the number of spikes is low and vice versa. Fear conditioning depressed IL excitability leading to decreased number of spikes and increased ISI, fAHP, and sAHP. Fear extinction reversed the depression between 4 and 24 hours after extinction. With the spontaneous return of fear, the number of spikes decreased and the ISI, fAHP, and sAHP increased returning IL excitability to depressed post-conditioning levels.

Overall our findings support the model shown in Figure 4B. In the naïve state, the outputs from the amygdala and IL are balanced. After fear conditioning, fear is driven by increased amygdala output and reduced IL inhibition. Initially after fear extinction, amygdalar plasticity reduces amygdala output to produce low fear. During the consolidation of fear extinction there is an increase in the intrinsic excitability of IL neurons leading to increased excitation of inhibitory ITC cells in the amygdala to subsequently inhibit CEA neurons and maintain reduced fear responses [20], [21], [24]. With the passage of time the intrinsic excitability of IL neurons reverts back to a depressed state leading to less activation of IL projections to the amygdala and the spontaneous recovery of fear. Compounds that prevent the decay in IL excitability could prolong the expression of extinction memory and be useful for improving treatment of patients with PTSD who have impaired extinction memory [16].

## Supporting Information

Data S1 **Raw data analyzed for this study.**
(XML)
